# Effects of *Fructus Psoraleae* Extract on the Intestinal Absorption Kinetics of Geniposide and Geniposidic Acid in Rat

**DOI:** 10.3390/molecules19067557

**Published:** 2014-06-06

**Authors:** Yan Huo, Yuxing Huang, Xiangmei Hou, Lifeng Han, Lei Wang, Erwei Liu, Xiumei Gao

**Affiliations:** 1 Tianjin University of Traditional Chinese Medicine, 312 Yuquan Road, Nankai District Tianjin, Tianjin 300193, China; 2 Tianjin Key Laboratory of Phytochemistry and Pharmaceutical Analysis, Tianjin University of Traditional Chinese Medicine, 88 Yuquan Road, Nankai District, Tianjin 300193, China; 3 Tianjin Key Laboratory of Chinese Medicine Pharmocology, Tianjin University of Traditional Chinese Medicine, 88 Yuquan Road, Nankai District, Tianjin 300193, China

**Keywords:** *Fructus Psoraleae*, *Cortex Eucommia*, compatibility, geniposide (GP), geniposidic acid (GPA), *in situ* intestinal perfusion

## Abstract

*Cortex Eucommia* has been used as a kidney-tonifying herbal medicine with a long history of compatibility with *Fructus Psoraleae*. Geniposide (GP) and geniposidic acid (GPA) are the two main chemical components in *Cortex Eucommia*. In the present study, the effects of *Fructus Psoraleae* extract (FPE) on intestinal absorption kinetics of GP and GPA in rat were investigated. Twenty four male Sprague-Dawley rats were randomly assigned into four groups which were treated with GP, GPA, GP mixed with FPE and GPA mixed with FPE, respectively, by *in situ* intestinal perfusion for 3 h. The samples of intestinal perfusion solutions were collected every 30 min, and analyzed by ultra high performance liquid chromatography (UPLC). The curves of time and residual quantities of GP and GPA (lnx) in the intestinal perfusion solution and the cumulative absorption rate were obtained. The results showed that FPE exhibited different effects on the intestinal absorption of GP and GPA in rat: it increased the intestinal absorption of GP (*p* < 0.05), while demonstrated no significant effect on the absorption of GPA.

## 1. Introduction

**Figure 1:**
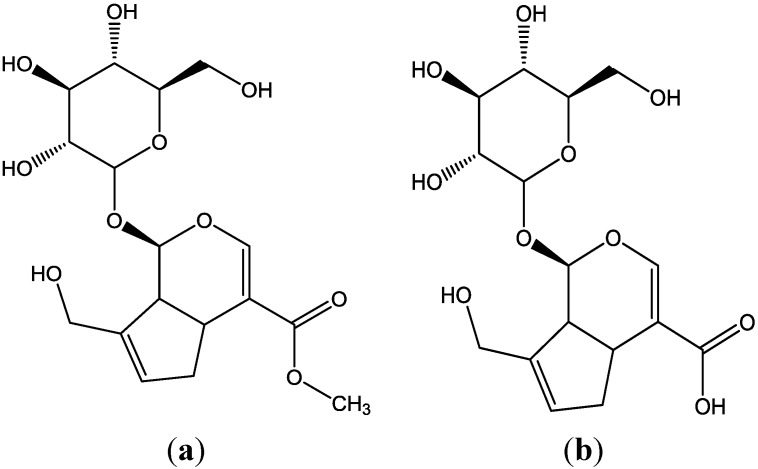
Structures of GP and GPA. (**a**) GP; (**b**) GPA.

*Cortex Eucommia*, is widely used in the clinic for the treatment of kidney or muscle weakness, lumbago and fetal irritability [1]. It is often administered together with *Fructus Psoraleae* extract (FPE) for kidney related diseases. GP and GPA (Figure 1) are iridoid compounds and active ingredients of *Cortex Eucommia* and many other Traditional Chinese Medicines [2,3,4,5]. Natural herbal medicines and their single ingredient compounds are widely used as clinical drugs, particularly in Asia. However, when administered simultaneously, the herbal medicine may significantly influence the absorption pharmacokinetics of the monomer compounds, thereby affect their effectiveness and safety. Several studies [6,7] have investigated the absorption kinetics of GP or GPA, but few studies have assessed the absorption kinetics of these two compounds combined with other Traditional Chinese Medicines. The effects of FPE on the intestinal absorption kinetics of GP and GPA in rat are still unknown, so it is very necessary to study the effects of *Fructus Psoraleae* extract on the intestinal absorption kinetics of GP and GPA in rat.

*In situ* intestinal perfusion is a widely used classic technique to evaluate drug absorption [8,9,10]. This method can reflect well the intestinal absorption of drugs because it does not involve cutting off blood vessels or nerves and the removal of the effects of gastric contents and physiological motions of digestive tracts. Therefore, it is the most suitable method to study the effects of FPE on the intestinal absorption kinetics of GP and GPA in rat.

UPLC as compared with HPLC produces better sensitivity, higher column efficiency and a wider flow range, so it provides good separation and can be used for complex system analyses [11,12,13]. This paper reports for the first time, the effects of FPE on the intestinal absorption kinetics of GP and GPA based on *in situ* intestinal perfusion and UPLC technologies.

## 2. Results

### 2.1. Chromatography

**Figure 2:**
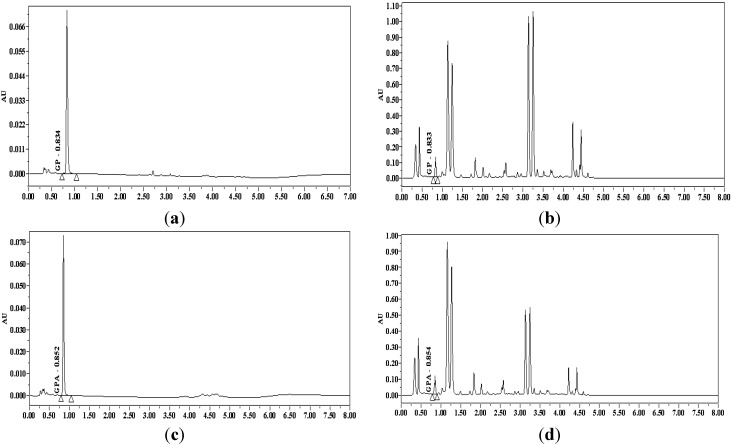
The chromatography of GP and GPA as single compounds and mixtures. (**a**) GP; (**b**) GP with FPE; (**c**) GPA; (**d**) GPA with FPE.

GP and GPA had a mean retention time of 0.85 min and 1.02 min respectively. Examples of the chromatographic results of single and compatibility groups are shown in Figure 2. As seen in the figure, GP and GPA were well separated both as single compounds single and in the mixtures.

### 2.2. Linearity

The calibration curves were linear in the range of 0.45–45 mg/L for GP and GPA without using a weight factor. The equations of standard curves were Y = 1.26 × 10^4^ X + 1.81 × 10^3^, R^2^ = 0.9997 (GP), Y = 5.72 × 10^2^ X + 5.43 × 10^3^, R^2^ = 0.9999 (GPA).

### 2.3. Stability

#### 2.3.1. Stability of GP and GPA in Blank Intestinal Perfusion Solution

A total of 50 mg/L GP and GPA perfusion solution (dissolved in blank intestinal perfusion solution) was incubated in a water bath at 37 °C for 3 h. The concentrations at 0, 0.25, 0.5, 1, 2 and 3 h were determined and compared. The results showed that both GP and GPA groups were stable in blank intestinal perfusion solution for 3 h (Table 1).

#### 2.3.2. Stability of GP and GPA in K-R Solution

GP and GPA were stable in K-R solution at room temperature for 24 h. The concentrations at 0, 1, 2, 3, 5, 6, 8, 10 and 24 h were determined and compared. The results were summarized in Table 1.

**Table 1:** Stability of GP and GPA.

Solutions	RSD (%)	
	GP	GPA
Blank intestinal perfusion solution (3 h)	3.27	0.45
K-R solution (24 h)	1.32	2.01

#### 2.3.3. Effect of Physical Adsorption of the Peristaltic Pump

The perfusion solutions with known drug concentrations were incubated for 3 h in a water bath at 37 °C. The concentrations of GP and GPA in the resulting solution were determined and compared with the original concentrations. The result showed physical adsorption of the peristaltic pump was negligible (Table 2).

**Table 2:** Effect of physical adsorption of the peristaltic pump.

	Concentration (mg/L)		Physical Adsorption
	**Original**	**3 h**	**RSD (%)**
GP	93.81	92.34	0.57
GPA	115.24	114.68	1.32

### 2.4. Recovery

The recoveries of GP and GPA ranged from 99.52% to 111.73% and 99.6% to 100.2% respectively (Table 3). It performed by analyzing those samples which made from drug-free blank intestinal perfusion solution by pre- and post- spiking into known standard and then processed to produce the concentrations at 0.5, 4 and 12 mg/L.

**Table 3:** Accuracy, precision and recovery of GP and GPA.

Level		Nominal (mg/L)	Recovery (Mean ± SD, %)	Accuracy (Deviation %)	Precision (RSD %)
GP	Low	0.5	99.52 ± 4.98	4.80	1.22	
	Mid	4	110.94 ± 2.04	3.98	0.87	
	High	12	111.73 ± 1.52	1.23	2.51	
GPA	Low	0.5	100.23 ± 0.66	6.1	2.62	
	Mid	4	99.78 ± 1.91	3.4	0.80	
	High	12	99.57 ± 0.87	1.4	0.12	

### 2.5. Accuracy, Precision and LLOQ

Accuracy and precision met the acceptance criteria suggested by the FDA, and were evaluated at the concentration levels of 0.5, 4 and 12 mg/L. The lower limit of quantification (LLOQ) of GP and GPA were 0.45 ng/mL and 0.5 ng/mL respectively. The results are listed in Table 3.

## 3. Discussion

We selected the duodenum to do the *in situ* intestinal perfusion because studies [14] have shown that the duodenum absorbed the GP better than other parts of the intestinal tract. In order to make a comparison with GP, we selected the same part for GPA.

Studies [15,16] have shown that compared with other concentrations, GP at 78 mg/L showed the best absorption in the duodenum. Therefore, we selected 80 mg/L as the concentration of the intestinal perfusion solution. The contents of GP and GPA in *Cortex Eucommia* extract were 0.279% and 0.754%. At the beginning, the concentration of GPA in the intestinal perfusion solution we selected was 216 mg/L (*i.e*., 80 × (0.754/0.279) mg/L), but it showed poor absorption. Then the concentrations of 40, 80 and 100 mg/L were also tested. It was noted that concentration of 80 mg/L appeared to be the most suitable concentration for GPA intestinal perfusion solution. According to the Chinese Pharmacopoeia, the dosage of *Cortex Eucommia* for adults is 6-10 g/day. Taking the extraction rate of *Cortex Eucommia* (13.5%, extracted in our lab) and the contents of GP and GPA in the *Cortex Eucommia* extract (0.279%, 0.754%) into account, the doses of GP and GPA for an adult were 2.3–3.8 mg/day and 6.1–10.2 mg/day respectively. In accordance with “TCM Pharmacodynamic Research and Evaluation” [17], the equivalent dose of GP and GPA for intestinal perfusion of 240 ± 20 g rats, calculated by the human and animal dose scaling method, should be 0.21–1.7 mg/day and 0.60–4.42 mg/day, respectively. The amount of GP and GPA we used in the perfusion solution was thus 2 mg. The dose of GP was a little beyond but close to the rational dosage, and the dose of GPA was in the rational dose range.

**Figure 3:**
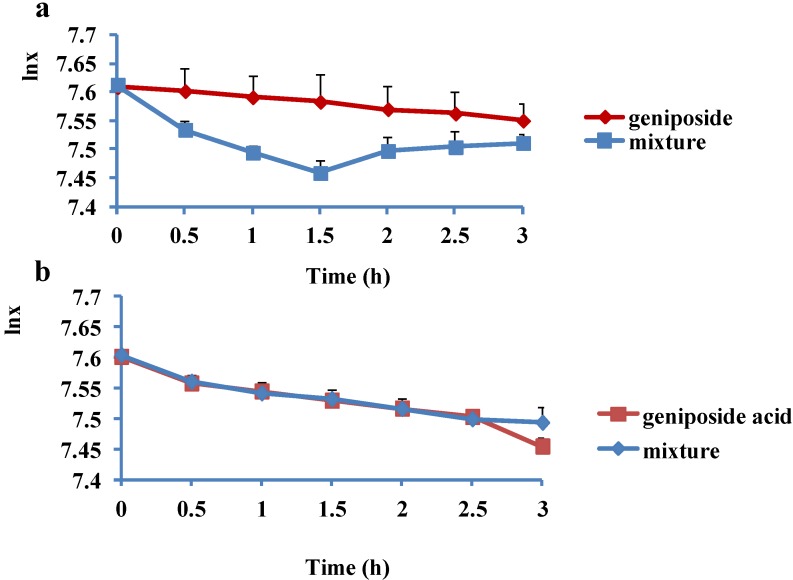
The curves of time and quantities (lnx) of GP and GPA in intestinal perfusion solution. (**a**) GP; (**b**) GPA.

The results (Figure 3, Table 4) showed that in a certain range of concentrations, FPE promoted the intestinal absorption of GP and affected the intestinal absorption kinetics of GP significantly in rat, especially after 1 h of adsorption. However, it had little effect on the intestinal absorption of GPA in rat. *Cortex Eucommia* is often mixed with *Fructus Psoraleae* for renal-related diseases, and a study by Liu [18] showed that GP inhibited NO production to protect from renal injury. This result provides biological evidence supporting the compatibility of GP and *Fructus Psoraleae* for renal diseases*.* The possible mechanism for the effects of FPE on the absorption kinetics of GP is the combination of the drugs and P-glycoprotein [16] (or enterohepatic circulation), however this needs further research for confirmation. An enzymatic study [19] showed that GP cannot be hydrolyzed by β-D-glucosidase in rat liver homogenate, but can be hydrolyzed by β-D-glucosidase of a human intestinal anaerobe, *Eubacterium* sp.A-44. It also can hydolyse GPA. Thus, the influence of FPE on the circumstances of enzyme or flora in the intestine may also affects the absorption kinetics of GP and GPA. Preliminary studies in our lab have shown that *Fructus Psoraleae* affected the *in vivo* exposure of ingredients of *Cortex Eucommia* to different degrees. We speculated that *Fructus Psoraleae* affects the absorption or metabolism of these compounds. Therefore we chose two markers; GP and GPA of *Cortex Eucommia* to conduct a study to explore the effects of *Fructus Psoraleae* on the absorption of these two components*.* The experimental results showed that FPE affected the absorption of GP, but had little effect on the intestinal absorption of GPA. Thus one can speculate that the effects of FPE on the absorption of GP may be one of the reasons that *Fructus Psoraleae* affects the *in vivo* exposure of GP.

**Table 4:** Cumulative absorption rate of GP and GPA (*n* = 6, * *p* < 0.05).

Component	Group	Cumulative Absorption Rate (%)	
		1 h (mean ± SD)	3 h (mean ± SD)
GP	Single	0.059 ± 0.018 *	0.081 ± 0.017 *
	Compatibility	0.139 ± 0.104	0.120 ± 0.063
GPA	Single	0.063 ± 0.040	0.136 ± 0.046
	Compatibility	0.075 ± 0.015	0.105 ± 0.028

## 4. Experimental

### 4.1. Reagents and Chemicals

GP and GPA (>98%) were obtained from Tianjin Science and Technology Party Co., Ltd. (Tianjin, China). Acetonitrile, methanol (HPLC grade) were purchased from Fisher Scientific Co. (Waltham, MA, USA). Acetic acid was obtained from Concord Chemicals Co., Ltd. (Tianjin, China). Ultra-pure water was prepared by using a Milli-Q system (Millipore, Bedford, MA, USA); FPE was prepared in our laboratory. KCl, CaCl_2_, NaHCO_3_ and NaH_2_PO_4_ (≥99% purity) were purchased from Tianjin North Medical Chemical Company (Tianjin, China). MgCl_2_, NaCl and glucose (re % purity) were obtained from Damao Chemical Company (Tianjin, China).

### 4.2. Instruments

The liquid chromatography system was a Waters Acqutity UPLC^®^ H-Class (SM-FTN injector, quaternary solvent pump, PDA detector). The UPLC column was a Waters BEH C18 column (2.1 × 50 mm, 1.7 µm) (both from Waters, Milford, MA, USA).

### 4.3. Preparation of Standard Solutions

#### 4.3.1. Krebs-Ringer’s Nutrient Solution (K-R Solution)

All the components were dissolved in the K-R solution to imitate the physiological conditions of the intestinal tract. K-R nutrient solution included: NaCl 7.8 g, KCl 0.35 g, CaCl_2_ 0.37 g, NaHCO_3_ 1.37 g, NaH_2_PO_4_ 0.02 g, MgCl_2_ 0.02 g, glucose 2.22 g. They were dissolved in distilled water to 1000 mL.

#### 4.3.2. Standard Solutions of GP and GPA

Standard solutions of GP and GPA were prepared by accurately weighting GP and GPA (1.50 mg) and dissolving in K-R solution and making up the volume to10 mL as the stock solutions respectively. The stock solution was sequentially diluted with pure water to make the concentrations of the working solution in the range of 0.45–45 mg/L in eight levels.

#### 4.3.3. Quality Control (QC)

QC samples were prepared at concentration levels of 0.5, 4 and 12 μg/mL. They were intermittently run with the test samples.

#### 4.3.4. Intestinal Perfusion Solutions

##### 4.3.4.1. Intestinal Perfusion Solutions of GP and GPA

Intestinal perfusion solutions of GP and GPA were prepared by weighing GP and GPA standards (4 mg) and dissolving in K-R solution in a graduated flask to 50 mL, respectively, and sonicating for 0.5 h.

##### 4.3.4.2. Intestinal Perfusion Solutions of Mixtures

FPE was prepared as follows: crude *Fructus Psoraleae* (200 g) was extracted with 70% (*v*/*v*) ethanol (1600 mL) under reflux twice, for 2 h each time. The solutions were combined and concentrated to dryness under vacuum at 50 °C. Finally, 27 g of FPE was obtained. The amount of *Fructus Psoraleae* that was mixed with GP or GPA was decided by the clinical combination (1:2), extraction rate of 70% ethanol (1:1) of *Fructus Psoraleae* and *Cortex Eucommia*, and the content of GP and GPA in the *Cortex Eucommia* extract (0.279%, 0.754%, analyzed by LC/MS/MS in our laboratory). The determined mixture of *Fructus Psoraleae* and GP was 179.2:1, 66.4:1 for GPA. The intestinal perfusion solutions of mixture were sonicated for 30 min before use*.*

##### 4.3.4.3. Blank Intestinal Perfusion Solution

The blank intestinal perfusion solution was prepared by a process consistent with the *in situ* intestinal perfusion experiment (as follows) except for the vacancy of GP or other components in the K-R solution.

#### 4.3.5. *In Situ* Intestinal Perfusion of GP and GPA

All animal experimental procedures were in compliance with the National Institutes of Health Guide for Care and Use of Laboratory Animals. The *in situ* intestinal perfusion experiment was performed by using male Sprague-Dawley rats (240 ± 20 g) which obtained from Beijing Vital River Laboratory Animal Technology Co., Ltd. (SCXK 2012-0001, Beijing, China). Animals were fasted overnight (12 h) with free access to water before being allocated to different experimental groups at random. After the abdominal cavity was opened, an intestinal loop (length, 10 cm) was made by cannulation with silicone tube (i.d., 3 mm) at the duodenum which was selected 1 cm away from pylorus. The intestinal contents were removed by infusion of normal saline solution. The 25 mL perfusion solution was precisely measured and stored in the measuring cylinder. At the start of the experiment, the perfusion solutions perfused through the intestinal segment at a flow rate of 5 mL/min for 10 min by using a peristaltic pump (Petro Gas Ausrüstungen, Berlin, Germany). On reaching a steady state, changed the flow rate to 2.5 mL/min, and the intestinal perfusion samples (150 µL × 3) were collected at 30 min intervals for the duration of 3 h (0, 0.5, 1, 1.5, 2, 2.5, 3 h) with a pipette and put into shell tube. After each collection, the volumes (V_1_) of the perfusion solutions were recorded precisely and 450 µL blank K-R solution was added into the perfusion solution to keep the volume of the perfusion solution constant. After 3 h, the intestinal perfusion solution in peristaltic pump and the duodenum was exhausted, the volume of which was recorded as V_2_. All the samples were assayed by UPLC after preparation. Finally, the curves of time and the residual quantities (lnx) of GP and GPA in intestinal solution during the absorption in the duodenum for 3 h were obtained (Figure 2).The residual quantities of GP and GPA in the perfusion solution were calculated using the following equation: X_n_ = C_n_V_n_ + 0.45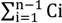, X_n_ is the residual quantities of GP or GPA in intestinal solution, C_i_ and C_n _is the concentration of the GP or GPA, V_n _is the volume of the intestinal perfusion solution (V_n_ = V_1_ + V_2_). Cumulative absorption rates were compared for 1 h and 3 h between the single and compatibility groups. The equation was as follows: 1 h/3 h cumulative absorption rate 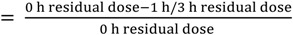. The data obtained was analyzed through independent samples T-test by SPSS, and was shown in Table 4.

### 4.4. Sample Preparation

Samples were prepared using a protein precipitation method at a volume ratio of 1:1 (*v*/*v*). The perfusion sample (150 µL) was mixed with CH_3_OH (150 µL), and then vortexed for 1 min. After 10 min for the precipitation, pure water (160 µL) was added to 40 µL of the above mixture. Then the sample was vortexed for 1 min and centr ifuged at 14,000 rpm for 10 min. The supernatant was directly used for UPLC analysis.

### 4.5. UPLC Conditions

After sample preparation, 3 µL of the supernatant was injected into the UPLC system, and separated on a Waters BEH C_18_ column (2.1 × 50 mm, 1.7 µm). The column temperature was set at 25 °C. The mobile phase was consisted of water with 0.2% acetic acid (A) and acetonitrile (B) at a flow rate of 0.35 mL/min. The mobile phase operated in the following gradient for GP: 0–3 min: 15%–50% B, 3–3.5 min: 50%–90% B, 3.5–4 min: 90%–50% B, 4–5 min: 50%–15% B; GPA: 0–1 min: 5%–10% B, 1–2 min: 10%–20% B, 2–3 min: 20%–50% B, 3–4 min: 50%–10% B, 4–5 min: 10%–5% B. The detection wavelength was 254 nm.

### 4.6. Data Analysis and Statistics

All UPLC data were acquired by the empower.3 workstation software (Waters Co., Milford, MA, USA). The concentrations (C) of GP and GPA in intestinal solution were observed with no interpolation.

## 5. Conclusions

A rapid and fully validated UPLC method was developed for the determination of GP, GPA and their mixtures with FPE after *in situ* intestinal perfusion. Through this study, we found that FPE demonstrated different effects on the intestinal absorption of these two similar compounds in rat. FPE promoted the intestinal absorption of GP and affected the intestinal absorption kinetics of GP significantly in rat. It prominently promoted the intestinal absorption of GP in the first 1 h, and had an influence on efflux in the following 2 h. Its influence on the intestinal absorption of GPA in rat was not obvious. This will play a guiding role in the combined use of Chinese herbal medicines and monomer compound drugs and provides scientific evidence for the clinical application and drug development of *Cortex Eucommia* and *Fructus Psoraleae.*

## References

[1] State Pharmacopoeia Commission (2010). Pharmacopoeia of People’s Republic of China.

[2] Venditti A., Serrilli A.M., Bianco A. (2013). Iridoids from Bellardia trixago (L.) All. Nat. Prod. Res..

[3] Qu K., Dai J., Zhao L., Lu Y., Li B., Zhao X., Hou P., Zhang Y., Bi K., Chen X. (2013). A sensitive liquid chromatographic-mass spectrometric method for simultaneous quantification of six iridoid glycosides from Zhi-zi-chi Decoction in rat plasma and its application to a pharmacokinetic study. J. Pharm. Biomed. Anal..

[4] Zhou Q., Lu W., Niu Y., Liu J., Zhang X., Gao B., Akoh C.C., Shi H., Yu L.L. (2013). Identification and quantification of phytochemical composition and anti-inflammatory, cellular antioxidant, and radical scavenging activities of 12 plantago species. J. Agric. Food Chem..

[5] Zhang Q., Su Y., Zhang J. (2013). Seasonal difference in antioxidant capacity and active compounds contents of Eucommia ulmoides oliver leaf. Molecules.

[6] Tan Q., Zhu J., Wang W., Wang Z., Cui J., Kong J., Qi M., Yang L. (2011). Study on absorption ingredients of Plantaginis semen by *in vitro* everted intestinal sac method. China J. Chin. Mater. Med..

[7] Du S., Zhang Q., Lu Y., Zhai Y., Wu Q. (2010). Study of components in xingnaojing affecting intestine absorption of gardenia extract. China J. Chin. Mater. Med..

[8] Almasi A., Bojcsev S., Fischer T., Simon H., Perjesi P., Fischer E. (2013). Metabolic enzyme activities and drug excretion in the small intestine and in the liver in the rat. Acta Physiol. Hung..

[9] Zhao G., Huang J., Xue K., Si L., Li G. (2013). Enhanced intestinal absorption of etoposide by self-microemulsifying drug delivery systems: Roles of P-glycoprotein and cytochrome P450 3A inhibition. Eur. J. Pharm. Sci..

[10] Patel J.R., Barve K.H. (2012). Intestinal permeability of Lamivudine using single pass intestinal perfusion. Indian J. Pharm. Sci..

[11] Yun H., Yan H., Zhang Z., Li J., Lu X., Liu X. (2013). [Determination of dicyandiamide, melamine and cyanuric acid in milk and milk powder by ultra performance liquid chromatography-tandem mass spectrometry]. Se Pu.

[12] Long Z., Zhang R., Zhao X., Meng X., Bi K., Chen X. (2013). Determination and pharmacokinetics of geniposidic acid in rat plasma after oral administration of Gardenia jasminoides fruit crude extract and Zhi-zi-chi decoction. Biomed. Chromatogr..

[13] Srikanth C.H., Chaira T., Sampathi S., V B.S., Bambal R.B. (2013). Correlation of *in vitro* and *in vivo* plasma protein binding using ultracentrifugation and UPLC-tandem mass spectrometry. Analyst.

[14] Zhang Q., Du S., Lu Y., Rao X. (2009). Studies on O/W partition coefficient and absorption kinetics of geniposide in fructus gardeniae extract in rat intestine. China J. Chin. Mater. Med..

[15] Zhai Y., Du S., Lu Y., Wang Y., Xu B., Gao Y. (2010). Impact of notoginseng total saponin on intestinal absorption kinetics of jasminoidin. China J. Tradit. Chin. Med. Pharm..

[16] Chula S., Hang L., Yinying B., Jianning S., Shi R. (2012). The effects of notoginsenoside R(1) on the intestinal absorption of geniposide by the everted rat gut sac model. J. Ethnopharmacol..

[17] Wang S.S.X., Huang X., Xie Y. (2005). TCM Pharmacodynamic Research and Evaluation.

[18] Liu E., Han L., Wang J., He W., Shang H., Gao X., Wang T. (2012). Eucommia ulmoides bark protects against renal injury in cadmium-challenged rats. J. Med. Food.

[19] Akao T., Kobashi K., Aburada M. (1994). Enzymic studies on the animal and intestinal bacterial metabolism of geniposide. Biol. Pharm. Bull..

